# CD9 promotes TβR2–TβR1 association driving the transition of human dermal fibroblasts to myofibroblast under hypoxia

**DOI:** 10.1186/s10020-024-00925-5

**Published:** 2024-09-27

**Authors:** Wanqi Huang, Ze Zhang, Xin Li, Qingqing Zheng, Chao Wu, Luojia Liu, Ying Chen, Jiaping Zhang, Xupin Jiang

**Affiliations:** Department of Plastic Surgery, State Key Laboratory of Trauma and Chemical Poisoning, Southwest Hospital, Army Medical University (Third Military Medical University), Chongqing, 400038 China

**Keywords:** Wound healing, Hypoxia, Myofibroblast, CD9, TGF-β1/Smad pathway, TβR2–TβR1 association

## Abstract

**Background:**

During wound healing, fibroblast to myofibroblast transition is required for wound contraction and remodeling. While hypoxia is an important biophysical factor in wound microenvironment, the exact regulatory mechanism underlying hypoxia and fibroblast-to-myofibroblast transition remains unclear. We previously found that tetraspanin CD9 plays an important role in oxygen sensing and wound healing. Herein, we investigated the effects of physiological hypoxia on fibroblast-to-myofibroblast transition and the biological function and mechanism of CD9 in it.

**Methods:**

Human skin fibroblasts (HSF) and mouse dermis wounds model were established under physiological hypoxia (2% O_2_). The cell viability and contractility of HSF under hypoxia were evaluated by CCK8 and collagen gel retraction, respectively. The expression and distribution of fibroblast-to-myofibroblast transition markers and CD9 in HSF were detected by Western blotting and immunofluorescence. CD9 slicing and overexpressing HSFs were constructed to determine the role of CD9 by small interfering RNA and recombinant adenovirus vector. The association of TβR2 and TβR1 was measured by immunoprecipitation to explore the regulatory mechanism. Additionally, further validation was conducted on mouse dermis wounds model through histological analysis.

**Results:**

Enhanced fibroblast-to-myofibroblast transition and upregulated CD9 expression was observed under hypoxia in vitro and in vivo. Besides, reversal of fibroblast-to-myofibroblast transition under hypoxia was observed when silencing CD9, suggesting that CD9 played a key role in this hypoxia-induced transition. Moreover, hypoxia increased fibroblast-to-myofibroblast transition by activating TGF-β1/Smad2/3 signaling, especially increased interaction of TβR2 and TβR1. Ultimately, CD9 was determined to directly affect TβR1–TβR2 association in hypoxic fibroblast.

**Conclusion:**

Collectively, these findings suggest that CD9 promotes TβR2–TβR1 association, thus driving the transition of human dermal fibroblasts to myofibroblast under hypoxia.

**Supplementary Information:**

The online version contains supplementary material available at 10.1186/s10020-024-00925-5.

## Introduction

Fibrosis, characterized by excessive collagen deposition and the transition of fibroblasts to myofibroblasts, can impact various organ systems, including the lungs, liver, kidney, and skin (Distler et al. 2019; Liu et al. 2022). While tissue organ fibrosis is concerning, the process of fibroblast to myofibroblast transition, which involves the upregulation of α-smooth muscle actin (α-SMA) proteins and the excessive synthesis of extracellular matrix (ECM) components like type I (COL-Ι) and type III fibrillar collagens (COL-Ш), is necessary for wound contraction and remodeling during the wound healing process (Rodrigues et al. 2019; Tai et al. 2021). Conversely, the insufficient transition of fibroblasts to myofibroblasts, consistent with less EMC deposition and delayed wound closure, is to blame for the suboptimal healing process (Tai et al. 2021). Transforming growth factor beta 1 (TGF-β1) is widely recognized for its involvement in the transition of fibroblasts to myofibroblasts and the formation of the EMC. During wound healing, TGF-β1 released at the site of wound directly binds to the type II receptors (TβR2) located on the fibroblast membrane, which then phosphorylate the cytoplasmic domain of the type I receptors (TβR1, Alk5) in a heterotetrameric receptor complex (Miyazawa and Miyazono 2017). The catalytically active TβRI phosphorylates the C-terminal serine residues of receptor-activated (R-) Smads named Smad2 and Smad3, which subsequently oligomerize with Smad4, forming trimeric protein complexes (Caja et al. 2018). These complexes are translocated to the nucleus, where they recognize specific Smad binding elements in the enhancer regions of α-SMA transcription causing enhanced deposition of ECM proteins (Carthy 2018). Although the classical TGF-β1/Smad pathway involved in fibroblast to myofibroblast transition has received significant attention, its regulatory mechanism in wound healing remains incompletely understood.

Wound healing is a highly intricate physiological process in the human body, involving three sequential but overlapping phases: inflammation, proliferation and remodeling, which can be regulated by biophysical microenvironment including mechanical pressure, endogenous electric fields, and temperature (Hinz et al. 2001; Rodrigues et al. 2019). One critical biophysical factor that can’t be ignored is oxygen which is required for almost every phase in wound healing process. Notably, a decline in oxygen levels at the wound site becomes evident as early as day 2 post-injury, reaching its peak on day 3, where the oxygen tension in the wounded tissue falls below 10 mmHg (Xing et al. 2011; Hong et al. 2014). That is, injured wounds undergo hypoxia because of the vascular damage and ascending oxygen consumption by activated cells surrounding the injured tissue during the initial stage of wound healing (Darby and Hewitson 2016). Correspondingly, hypoxia is shown to initiate inflammation phase by recruiting functional cells upon incisions and promotes angiogenesis, vasculogenesis and granulation tissue development in later proliferation stage until oxygen levels normalize (Schreml et al. 2010; Tirpe et al. 2019). Recent studies have also indicated that hypoxia may play a role in regulating collagen deposition and the transition of fibroblasts to myofibroblasts at the wound margin (Zhao et al. 2017; Leinhos et al. 2019). Furthermore, it has been observed that the signaling of TGF-β1 is intricately associated with the hypoxic microenvironment, leading to the induction of tumor epithelial-mesenchymal transition (EMT) and fibrosis in a manner dependent on TGF-β1 (Mallikarjuna et al. 2022). Therefore, we hypothesize that early physiological hypoxia is the driving factor for fibroblast-to-myofibroblast transition involving TGF-β1/Smad pathway.

CD9 is a member of the tetraspanin superfamily that regulates numerous cellular processes such as cell migration, proliferation, and transition (Brosseau et al. 2018). It is widely expressed and consists of four transmembrane domains, an intracellular terminus, and two extracellular loops. CD9 acts as an organizer of surface multiprotein complexes by associating with specific proteins such as TβR1 and TβR2, thereby enhancing their activities in diverse cellular processes, including the TGF-β1/Smad pathway, as evidenced in tumor studies (Wang et al. 2015; Lorico et al. 2021). However, there is limited research on the mediating mechanisms underlying the involvement of CD9 and the TGF-β1/Smad pathway in the process of wound healing. According to our previous study, delayed wound repair was observed in CD9-knockout mice, suggesting that tetraspanin CD9 had a crucial impact on wound healing process (Zhang et al. 2012). Further researches demonstrate that CD9 promotes wound healing by regulating the migration and transition of cells (Jiang et al. 2020). Moreover, CD9 expression can be regulated by oxygen tension and mediates hypoxic-induced cellular activity (Jiang et al. 2014). Thus, CD9 may mediate hypoxia-induced fibroblast-to-myofibroblast transition involving TGF-β1/Smad pathway.

Here, using HSFs and mouse dermis wounds as models, we identified a role for hypoxia in regulating fibroblast-to-myofibroblast transition and CD9 expression. Our findings demonstrated that hypoxia induced fibroblast-to-myofibroblast transition along with upregulated CD9 expression and CD9 overexpression accelerated hypoxic fibroblast transition, while CD9 silencing reversed fibroblast-to-myofibroblast transition induced by hypoxia. Furthermore, we elucidated that hypoxia activated the TGF-β1/Smad2/3 signaling pathway and that CD9 directly affects TβR1–TβR2 association in hypoxic fibroblast. Collectively, our findings indicate that tetraspanin CD9 may be involved in the fibroblast-to-myofibroblast transition induced by hypoxia through modulation of TGF-β1/Smad signaling pathway, where CD9 directly regulated the interaction of TβR1 and TβR2. These results contribute new insights into fibroblast-to-myofibroblast transition involving TGF-β1/Smad pathway during early physiological hypoxia and mechanisms of tetraspanin CD9 regulation in wound healing.

## Materials and methods

### Ethics statement

C57 mice (male, about 25 g) used in the experiment were provided from the Experimental Animal Department of the Army Medical University in Chongqing, China. The entire project was reviewed and approved by the Animal Experiment Ethics Committee of the Army Medical University. All animal-based investigations were designed and conducted in accordance with the Guide for the Care and Use of Laboratory Animals published by the National Institutes of Health (NIH Pub. No. 85-23, revised 1996).

### Human skin fibroblast and human keloid fibroblast cultures

Human skin fibroblasts (lot no. AC338126) and Human keloid fibroblasts (lot no. CP-H235) were obtained from Cell Bank of the Chinese Academy of Sciences in Beijing, China and were maintained in Dulbecco’s modified Eagle’s medium (C11995500BT, Gibco, Canada) including 10% fetal bovine serum (S-FBS-500, Scitecher, USA) and 1% penicillin streptomycin (GA3502, Genview, Australia). Cell cultures were performed in a 5% CO_2_ atmosphere at 37 °C. The medium was changed three times a week. When the culture reached 90% confluence, the cells were separated from the flask with 0.05% trypsin-0.1% ethylenediaminetetraacetic acid (EDTA) solution, washed twice, and then resuspended in DBS supplemented with FBS medium. In each experiment, fibroblasts were used between passages 4 and 5.

### Hypoxia exposure

Cells were exposed to normoxia (21% O_2_) in a normal CO_2_ cell culture incubator or hypoxia (2% O_2_) in a hypoxia chamber (Billups-rothenburg, Del Mar, CA). Sterile water was placed inside the chamber to maintain moist conditions. Hypoxic conditions were created by filling the chamber with 2% O_2_, 5% CO_2_ and balanced N_2_. The gas was passed through the chamber at 1–2 psi for 3 min, and the chamber was sealed and placed in a 37 °C cell culture incubator. The oxygen concentration in the chamber was monitored using an oxygen sensor (OxyCheq, Marianna, FL). Every 2 days, media were replaced with fresh media that were degassed using a SpeedVac Concentrator system (Termo Fisher Scientifc, Waltham, MA).

### Cell viability

Cell viability was determined using a cell counting kit-8 assay (Dojindo, Kumamoto, Japan). Briefly, cells were cultured in 96-well plates at a density of 2000 cells/well and exposed to normoxia or hypoxia for 12 h, 24 h or 48 h and cell viability was detected according to the manufacturer’s instructions. The absorbance at 450 nm was measured by a multidetection microplate reader (Model: Synergy 2; BioTek Instruments Inc, Winooski, VT).

### Collagen gel retraction

Rat tail collagen was extracted and purified before DMEM was added to the rat tail collagen as previously described (Suttho et al. 2017). After the color of the mixture changed to golden yellow, 1 M NAOH was slowly added until the color changed to red, and then, 50,000 HSFs were inoculated in 200 ml collagen mixture, mixed, and added to a 24-well plate for 1 h. After collagen coagulation, HSFs were cultured under hypoxia. After 12 h, 24 h and 48 h of culture, three-dimensional collagen was separated from the side walls and photographed to measure the gel contour. The ratio of the gel profile to the well profile was used as a measure of shrinkage strength.

### Protein extraction and western blot analysis

Total proteins from cells were extracted using RIPA lysis buffer containing phosphatase and protease inhibitors (Beyotime Biotechnology). The concentration of total protein was detected with a BCA Protein Assay kit (Beyotime Biotechnology). Equal amounts (30 μg) of protein were then separated using 4–20% sodium dodecyl sulfate polyacrylamide gel electrophoresis gels. Proteins were then transferred to nitrocellulose membranes. The membranes were blocked with 5% non-fat milk in Tris-buffered saline, and incubated with primary antibodies. Overnight at 4 °C and incubated with the corresponding secondary antibody at room temperature for 1 h. The molecular imager ChemiDoc TMXRS + imaging system (Bio-Rad) and chemiluminescent reagents detected the signal together. Western blot band intensities were quantified using ImageJ. The using of primary antibodies was as follows: α-SMA (1:1000, ab32575, Abcam, UK), COL-1 (1:1000, ab90395, Abcam, UK), COL-3 (1:1000, 22734-1-AP, Proteintech, USA), GAPDH (1:5000, HRP-60004, Proteintech, USA), CD9 (1:1000, ab92726, Abcam, UK), Smad2/3 (1:1000, ab202445, Abcam, UK), pSmad2/3 (1:1000, #8828S, Cell Signaling Technology, US), TβR1 (1:1000, ab230788, Abcam, UK), TβR2 (1:1000, ab159745, Abcam, UK).

### Immunofluorescence and confocal analysis

Hypoxia induced HSFs cultured on fibronectin-coated glass covers lips were treated as above, then fixed in 4% paraformaldehyde for 20 min. After 0.5% Triton X-100 permeabilization for 10 min and 5% goat serum in PBS blocking for 1 h, HSFs were incubated with mouse anti-α-SMA or anti-CD9 (1:100 dilution) at 4 °C overnight, washed with PBS and followed by incubation with Alexa Fluor 568 conjugated secondary antibody at 37 °C for 1 h. Nuclei were stained with DAPI (Hyclone, USA). The α-SMA and CD9 expression were observed under Leica Confocal Microscope (Leica Microsystems, Wetzlar, Germany).

### Recombinant adenovirus vector to overexpress CD9 expression

The recombinant adenovirus vectors for CD9 overexpressing (Ad-CD9-GFP) and the negative control adenovirus vectors encoded the GFP sequence (vector) were purchased from Shanghai GeneChem, Co. Ltd (Shanghai, China). Vectors contained the gene for GFP, which served as a marker. HSFs were infected with these vectors at a multiplicity of infection of 10 for 48 h for further experiments.

### Small interfering RNA transfection

To knockdown CD9 in HSFs, a pool of siRNAs for the CD9 (sc-35032) gene and non-specific control siRNAs (sc-35032-PR) were purchased from Santa Cruz Biotechnology (Carlsbad, CA). Second passage HSFs were transfected with small interfering RNA (siRNA) for CD9 or negative control according to the manufacturer’s protocol.

### Histological analysis

A 3-mm-diameter full-thickness wound was punched on the dorsal midline of 8-week-old C57 male mice using a biopsy punch. Wounded areas surrounded by unwounded skin were dissected at day 0, 1 and 3 after injury, fixed in paraformaldehyde and embedded in paraffin. For Immunofluorescence (IF), sections were performed by heating to 95 °C in 0.01 M of citrate buffer to retrieve antigen. Sections were blocked in 10% normal goat serum in PBS for 1 h in a humidified atmosphere at 37 °C. Subsequently, sections were incubated overnight at 4 °C in primary antibodies: anti-CD9, anti-Cytokeratin 10 (1:100 dilution; Santa Cruz, USA), then washed three times with PBS and incubated for 1 h with Alexa Fluor 488 or 568 secondary antibodies (1:100 dilution; Invitrogen, USA). After two washes in PBS, the tissue sections were counter stained with DAPI (Hyclone, USA) to highlight nuclei. Fluorescence was observed using a Leica confocal microscope (Leica Microsystems, Wetzlar, Germany). For immunohistochemistry (IHC), sections were incubated overnight with primary antibodies against p-smad2/3 (1:3000, Thermofisher). After dewaxing and being closed in 5% goat serum for 1 h at room temperature. After washing in PBS, secondary antibodies were added for 2 h (1:100 dilution) and finally an appropriate amount of 3,3ʹ-diaminobenzidine (DAB) was dropped in for color development. After taking pictures under the microscope, the results were analyzed by Image-J.

### Immunoprecipitation (IP)

HSFs underwent cytolysis in 1 ml RIPA buffer, followed by 10 min incubation on ice. Total cell lysates accepted 10 min centrifugation at 10,000×*g*, at 4 °C. Then the supernatants received 30 min incubation using 20ul protein A/G PLUS-agarose (Santa Cruz sc-2003) at 4 °C. Pellet beads received 5 min centrifugation at 2500 rpm, at 4 °C. 10 ul of primary antibody was used to incubate the supernatants for 60 min. The complexes were then precipitated through the addition of 20 ul protein A/G PLUS- agarose into the lysate, followed by being incubated at 4 °C for overnight. The 30 s centrifugation on beads was performed at 2500 rpm, at 4 °C, then ice-cold RIPA buffer was employed to wash the beads four times. The samples were then suspended and denatured in SDS sample buffer (which contained 100 mM dithiothreitol, 10% glycerol, 50 mM Tris pH 6.8, 2% SDS, and 0.01% bromophenol blue).

### Statistical analysis

The statistical analyses were performed using GraphPad Prism version 8.0 (GraphPad Software, San Diego, CA). Data are represented as mean ± standard error of mean (SEM). In order to compare the statistical differences between any pair of data, t-tests and one-way ANOVA test were used to calculate the p-value. p < 0.05 was considered significant.

## Results

### Hypoxia induces fibroblast-to-myofibroblast transition and contraction in dermal fibroblasts

**Fig. 1:**
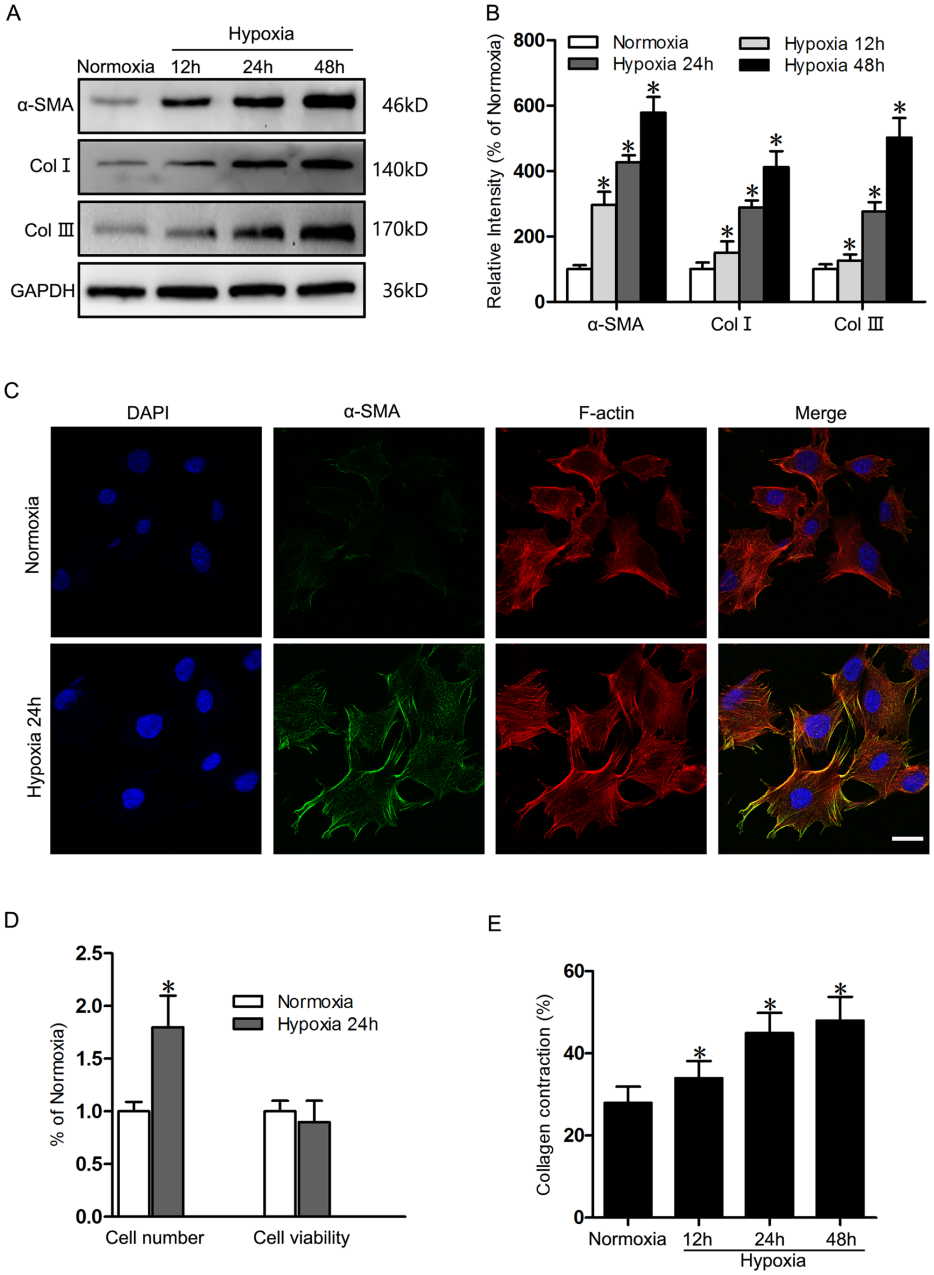
Hypoxia induces fibroblast-to-myofibroblast differentiation and contraction in HSFs. **A** Western blot was used to detect α-SMA, COL-1 and COL-3 in HSFs under normoxic and hypoxic conditions (after 12, 24, 48 h of hypoxia). GAPDH was monitored as a gel-loading control. **B** The results were quantified by relative intensity. **C** Immunofluorescence staining of α-SMA and F-actin on HSFs under normoxia and hypoxia for 24h. Bar = 20μm. **D** Cell number was quantified by cell counting and viability was assessed using a CCK8 cell viability assay. **E** Effect of hypoxia on collagen gel contraction. Cells grown in collagen gels were incubated under normoxic and hypoxic conditions (after 12, 24, 48 h of hypoxia). Gels were lifted and changes in collagen areas were determined over a time period of 24 h. For each condition 4 collagen gels were analyzed. Data are the mean ± SEM of three independent experiments performed in triplicate. *, p < 0.05 versus Normoxia group

Hypoxia is a common phenomenon in phases of wound healing, and it can significantly affect the wound microenvironment (Wang et al. 2021). During wound healing, fibroblasts transdifferentiate to α-SMA-positive myofibroblast which are responsible for excessively ECM-producing and wound contraction (Tai et al. 2021). To explore the effect of hypoxia on fibroblast-to-myofibroblast and collagen deposition, cultured HSFs were then exposed to hypoxic conditions (2% O_2_). In hypoxia, α-SMA protein expression increased significantly in a time-dependent manner, with 2.9-, 4.2-, and 5.8-fold increase after 12 h, 24 h and 48 h hypoxia respectively. COL-Ι and COL-Ш which partly reflected the collagens secreted by myofibroblasts, also increased 1.5- and 1.3-fold in 12 h hypoxia, 2.9- and 2.7-fold in 24 h hypoxia, and 4.1- and 4.1-fold in 48 h hypoxia (Fig. 1A, B). These upregulation in fibroblast-to-myofibroblast transition were further supported by immunofluorescence staining. The number of α-SMA-positive cells in which α-SMA colocalizes with F-actin increased significantly when cells were cultivated in hypoxic conditions for 24 h (Fig. 1C). An obvious increase of fibroblast cell number by 1.8-fold and a slight decrease of viability by 8% was observed in 24 h hypoxia (Fig. 1D). Since α-SMA proteins is responsible for the generation of contractile force in myofibroblasts, the ability to contract collagen matrices is thus another characteristic of myofibroblast transition. Fibroblasts were therefore cultured in a polymerized collagen matrix in normoxia or hypoxia, then the matrices were released from the culture dish, resulting in mechanical unloading and contraction driven by the force generated by the cells. When hypoxia for 12 h, 24 h and 48 h, the contraction of the collagen matrices by fibroblasts in low oxygen concentration was 21.4%, 60.1% and 71.4% higher than contraction in normoxic conditions (Fig. 1E).

### Hypoxia induces CD9 expression in fibroblast cells

**Fig. 2:**
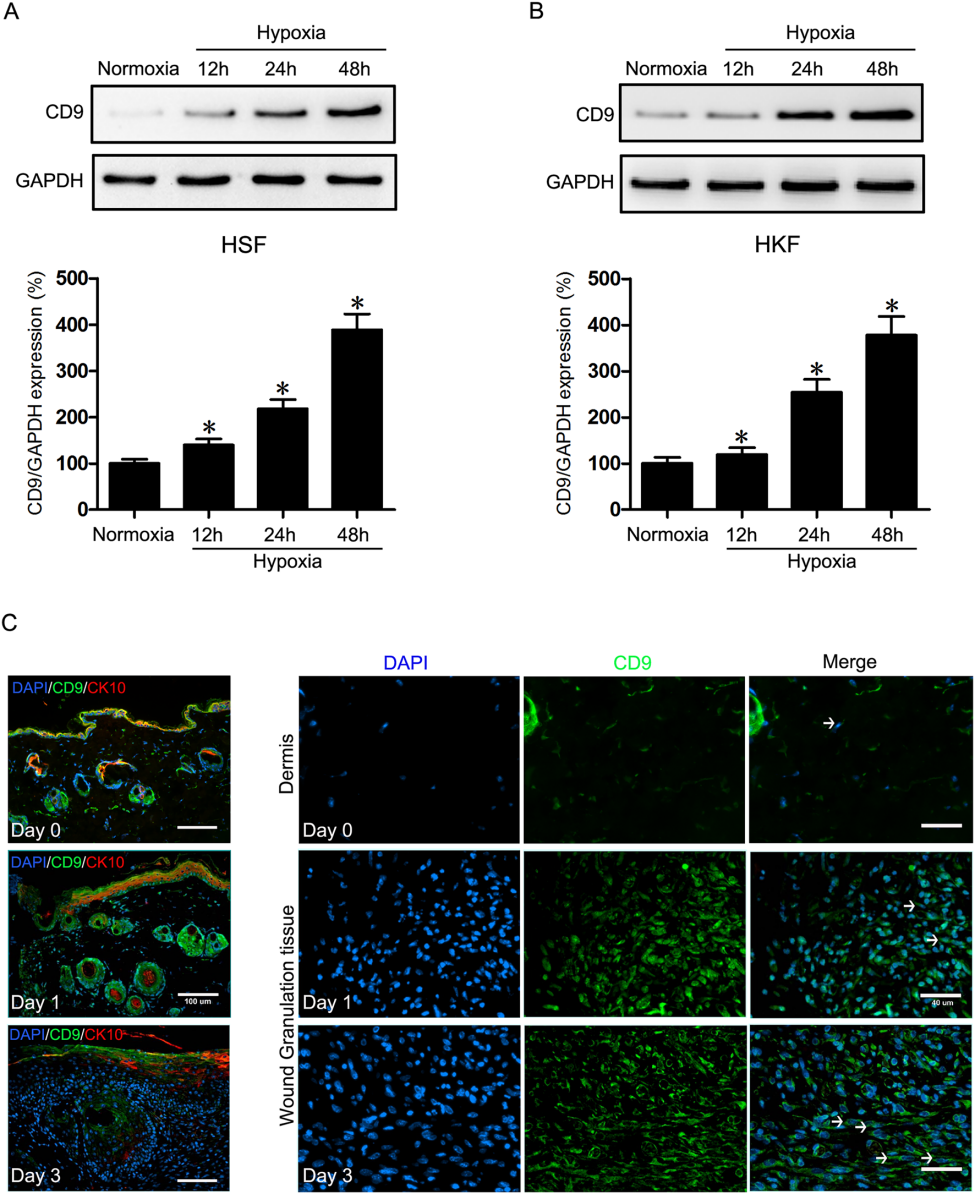
Hypoxia induces CD9 expression in HSFs and HKFs. **A**, **B** Western blot was used to detect CD9 in HSFs and HKFs under normoxic and hypoxic conditions (after 12, 24, 48 h of hypoxia). GAPDH was monitored as a gel-loading control. The results were quantified by relative intensity. **C** Immunofluorescence staining of CD9 and differentiation marker cytokeratin 10 in normal unwounded skin (day 0), day 1 and day 3 wound sections obtained from mice showing CD9 was higher in early hypoxic microenvironment during physiological wound healing. Narrow-dotted line: differentiated dermal HSFs. Bars: left column 100μm, right column 40μm. Data are the mean ± SEM of three independent experiments performed in triplicate. *, p < 0.05 versus Normoxia group

Tetraspanin CD9, has been implicated in a wide variety of cellular biological processes such as cell motility, adhesion, and transition (Oritani et al. 2000; Castilho et al. 2013; Orenstein 2014). For evaluating the possible connection between hypoxia and CD9 on HSFs and HKFs (Human keloid fibroblasts), Western blots were performed after cultured cells were then exposed to hypoxic conditions (2% O_2_). HSFs showed separate 1.4-, 2.2- and 3.9-fold increase of CD9 protein expression along with prolongationtime in hypoxic microenvironment. Similarly, the same effect on CD9 protein expression was observed in HKFs, accompanied by 1.2-, 2.6- and 3.9-fold rise in 12 h, 24 h and 48 h hypoxia (Fig. 2A, B). On close examination of the intercellular distribution of CD9 at early hypoxic stage in vivo, we constructed a wounded mice model with 3-mm-diameter full-thickness wounds on the dorsal midline and dissected wounded areas on day 0, 1 and 3 since it has been reported that hypoxia lasts more than 3 days in wounded skin. Then double immunofluorescence staining was performed on CD9 and cytokeratin 10 (CK10) before the confocal microscopy analysis. The number of CD9-positive cells increased significantly in wound granulation tissues dissected on day 1 and 3 compared with dermis (Fig. 2C), which indicated an obvious uptrend of CD9 expression in the early stage of wound healing where hypoxia played a prominent role in microenvironment. More specifically, CD9 was confined to the suprabasal layers expressing CK10 at a relative low level immediately after the wound (Day 0, Fig. 2C). However, in wound granulation tissues on day 1 and 3, CD9 expressed at an ascending level in the stratum basale (low cytokeratin 10 expression area, day 1 and 3, Fig. 2C). These results indicated that hypoxia induced fibroblast cells to express CD9, which was further supported by animal wound models.

### Effect of CD9 on hypoxia-induced fibroblast-to-myofibroblast transition

**Fig. 3:**
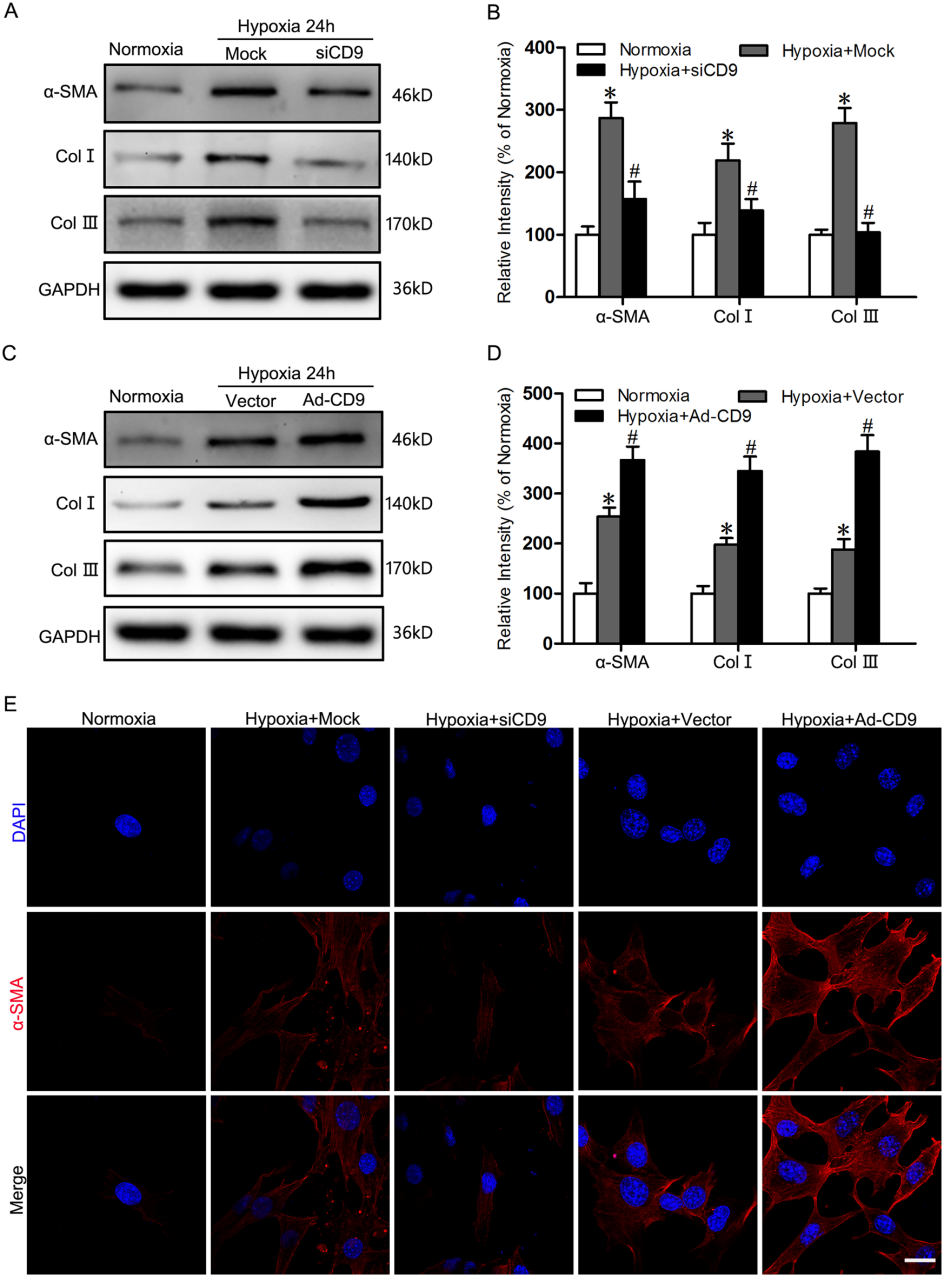
Effect of CD9 on hypoxia-induced fibroblasts-to-myofibroblast Differentiation. **A** Western blot was used to detect expression of α-SMA, COL-1 and COL-3 in CD9 silence HSFs under normoxia and hypoxia for 24h. **B** The results were quantified by relative intensity. The data was shown as the mean ± SEM (n = 3). *, p < 0.05 versus Normoxia group; #, p < 0.05 versus Hypoxia + Mock group. **C** Western blot was used to detect expression of α-SMA, COL-1 and COL-3 in CD9 overexpression HSFs under normoxia and hypoxia for 24 h. **D** The results were quantified by relative intensity. The data was shown as the mean ± SEM (n = 3). *, p < 0.05 versus Normoxia group; #, p < 0.05 versus Hypoxia + Vector group. **E** Immunofluorescence staining of α-SMA and F-actin on HSFs under normoxia and on CD9 silence or over-expression HSFs under hypoxia for 24 h. Bar = 20μm

To see if CD9 indeed plays a role in fibroblasts-to-myofibroblast transition, we performed a set of experiments in HSF cells. First of all, we knockdown CD9 on HSFs under culture by on-target siRNA to evaluate whether silencing CD9 restricts the transition of fibroblasts. Then they were exposed to hypoxic conditions (2% O_2_) for 24 h before Western blots were constructed. The expression of α-SMA decreased by 45.2% in CD9-silenced HSFs (p > 0.05) compared with the mock group in 24 h hypoxia. Moreover, collagens deposition induced by hypoxia also suppressed by silencing CD9, since protein expression of COL-Ι and COL-Ш decreased by 36.5% and 62.7% in siCD9 group (Fig. 3A, B). By contrast, cultured in hypoxic environment for 24 h, stably overexpressing CD9 HSFs constructed by recombinant adenovirus vectors showed 44.4% increase of α-SMA protein expression compared with the mock group (p > 0.05). Similarly, COL-Ι and COL-Ш in CD9-overexpressing HSFs obviously ascended by 74.2% and 1.04-fold respectively, which indicated that highly expressed CD9 further induced collagen deposition in the hypoxic microenvironment (Fig. 3C, D). The expression and distribution changes of α-SMA, COL-Ι and COL-Ш were further supported by immunofluorescence staining when silencing or overexpressing CD9 in hypoxia. After cultured in hypoxia for 24 h, the number of α-SMA-positive cells descended significantly when CD9 was knocked down, while ascended obviously in CD9-overexpressing HSFs (Fig. 3E). These results suggested that CD9 played a key role in inducing fibroblast-to-myofibroblast transition in hypoxia.

### Hypoxia activates TGF-β1/Smad2/3 signaling and increases the interaction of TβR2 and TβR1 in fibroblasts

**Fig. 4:**
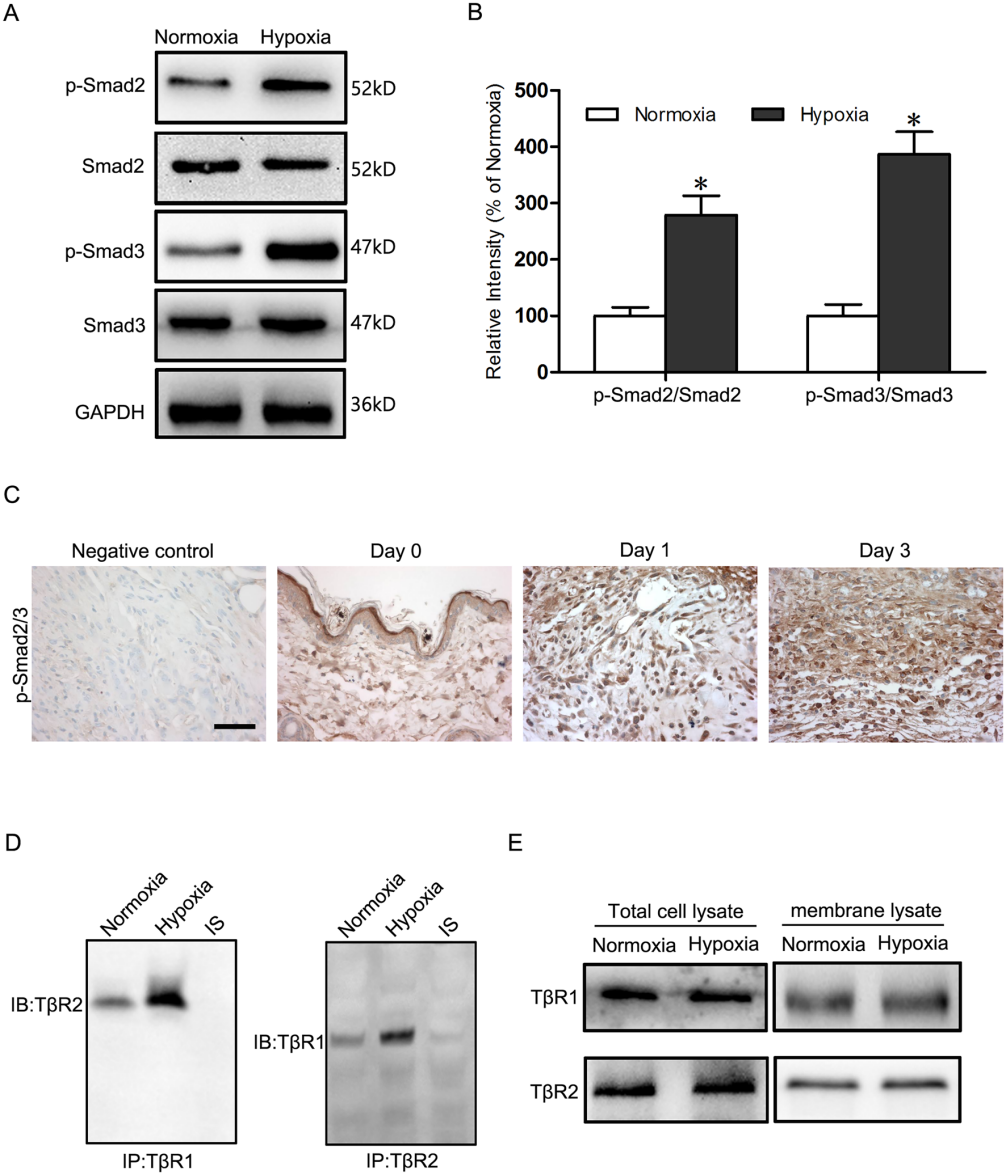
Hypoxia activates TGF-β1/Smad2/3 Signaling and increases the interaction of TβR2 and TβR1 in HSFs. **A** Western blot was used to detect expression of p-Smad2, Smad2, p-Smad3 and Smad3 in HSFs under normoxia and hypoxia for 24h. GAPDH was monitored as a gel-loading control. **B** The p-Smad2/Smad2 and p-Smad3/Smad3 were quantified by relative intensity. The data was shown as the mean ± SEM (n = 3). *, p < 0.05 versus Normoxia group. **C** Immunofluorescence staining of p-Smad2/3 in normal unwounded skin (negative control), day 0, day 1 and day 3 wound sections obtained from mice showing p-Smad2/3 was highest in day 3. Bar = 50 μm. **D** Immunoprecipitation analysis of the association between TβR2 and TβR1 in HSFs under normoxia and hypoxia for 24h. **E** The expressions of TβR1 and TβR2 in total protein and membrane protein under normoxia and hypoxia for 24 h

TGF-β1/Smad signaling is a pivotal fibrogenic factor that is responsible for fibroblast-to-myofibroblast transition and excessive ECM deposition (Mingyuan et al. 2018; Tai et al. 2021). Emerging evidence has indicated that biophysical microenvironment (such as mechanical stress, fluid shear stress, and wound hypoxia) regulate fibroblast transition and fibrosis through activated TGF-β1/Smad signaling. Therefore, we investigated whether TGF-β1/Smad2/3 Signaling is promoted in hypoxic microenvironment. Before Western blots targeting on Smads were constructed, HSFs were exposed to hypoxia (2% O2) for 24 h. The expression of p-Smad2/Smad2 and p-Smad3/Smad3 that represents activated TGF-β1/Smad2/3 signaling increased 2.8- and 3.9-fold separately, compared with the normoxia (p > 0.05) (Fig. 4A, B). Moreover, the results were validated in animal models where p-Smad2/3 was detected by immunohistochemistry. It has been reported that hypoxia lasts more than 3 days in wounded skin, so a wounded mice model with 3-mm-diameter full-thickness wounds was constructed on the dorsal midline and dissected on day 0, 1 and 3. As expected, the level of p-Smad2/3 ascended gradually in early hypoxic microenvironment and showed a remarkable increase compared with negative control (Fig. 4C). To determine the underlying mechanism behind hypoxia-activated TGF-β1/Smad signaling, we performed Immunoprecipitation with anti-TβR1 and anti-TβR2 antibody respectively, followed by Western blot analysis and to detect the interaction of TβR2 and TβR1 in fibroblasts. Hypoxia increased the association between TβR1 and TβR2 in HSFs compared with normoxia (Fig. 4D). The expressions of TβR1 and TβR2 in total protein and membrane protein showed no difference between normoxia and hypoxia groups, suggesting that hypoxia has no effect on the expression and distribution of TβR1 and TβR2 in cell or membrane, which further proved that hypoxia promoted the interaction between TβR1 and TβR2 (Fig. 4E).

### CD9 directly affects TβR1–TβR2 association in hypoxic fibroblast

**Fig. 5:**
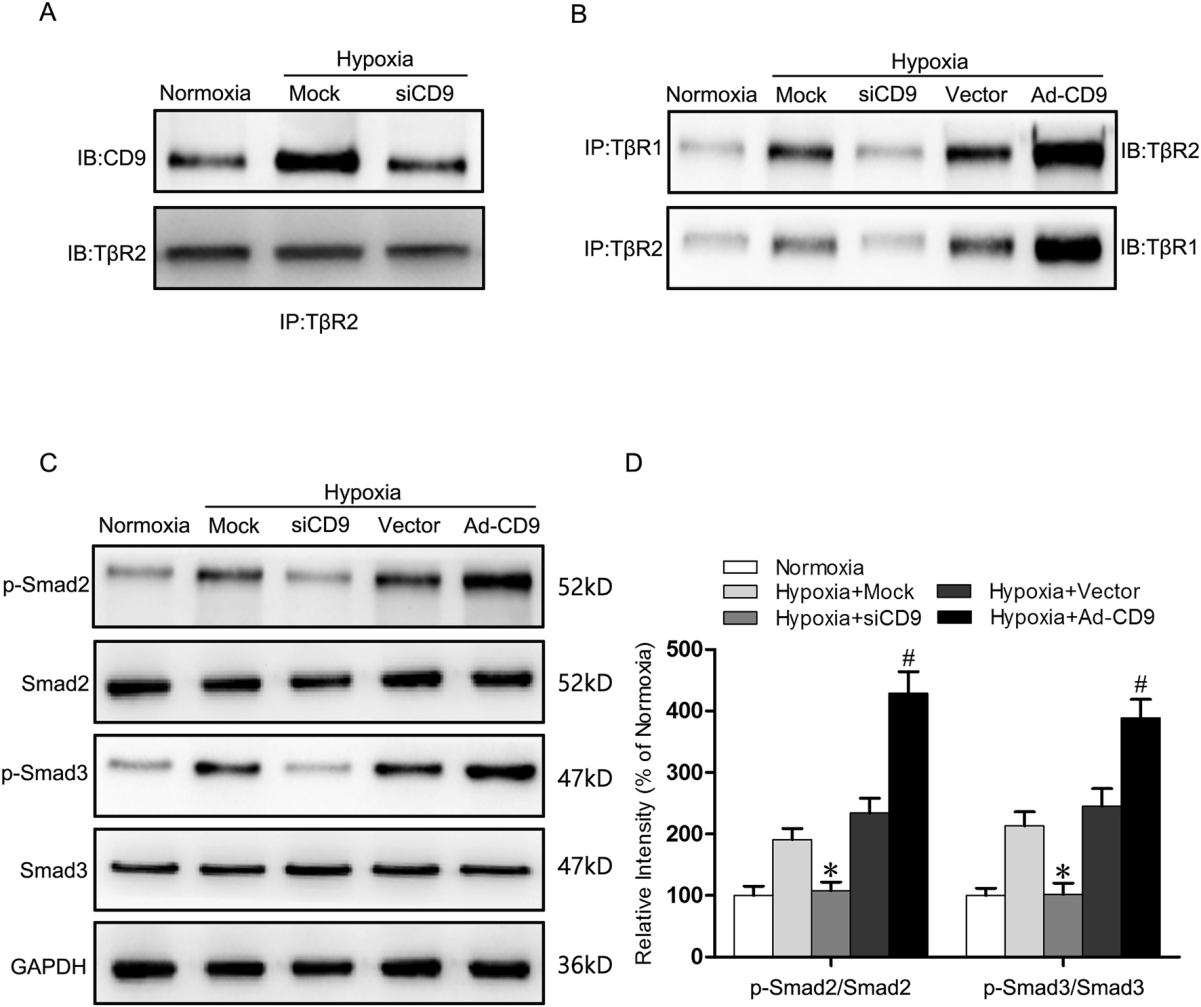
CD9 directly affects TβR1–TβR2 association in hypoxic HSFs. **A** Immunoprecipitation analysis of the association between TβR2 and TβR1 in HSFs under normoxia and in CD9 silence HSFs under hypoxia for 24 h. **B** Immunoprecipitation analysis of the association between TβR2 and TβR1 in HSFs under normoxia and in CD9 silence or over-expression HSFs under hypoxia for 24 h. **C** Western blot was used to detect expression of p-Smad2, Smad2, p-Smad3 and Smad3 in HSFs under normoxia and in CD9 silence or over-expression HSFs under hypoxia for 24 h. GAPDH was monitored as a gel-loading control. **D** The p-Smad2/Smad2 and p-Smad3/Smad3 were quantified by relative intensity. The data was shown as the mean ± SEM (n = 3). *, p < 0.05 versus Hypoxia + Mock group; #, p < 0.05 versus Hypoxia + Vector group

To determine whether the increased TβR1–TβR2 association in HSFs may account for CD9-mediated fibroblast-to-myofibroblast transition under hypoxia, we transfected HSFs with siRNA to silence CD9 (siCD9) and constructed recombinant adenovirus vectors to overexpress CD9 (Ad-CD9). Then immunoprecipitation with anti-TβR2 antibody was performed, followed by Western blot analysis on CD9 and TβR2, which demonstrated that hypoxia increased the association between CD9 and TβR2 compared with normoxia while silencing CD9 decreased that (Fig. 5A). Moreover, we carried out Immunoprecipitation with anti-TβR1 and anti-TβR2 antibody respectively and Western blot analysis on TβR2 and TβR1. Overexpressing CD9 increased the association between TβR1 and TβR2 compared with simple hypoxia but decreased TβR1–TβR2 association was observed in silencing CD9, suggesting that TβR1–TβR2 association was directly affected by CD9 (Fig. 5B). In addition, Western blots targeting on Smads were constructed in HSFs under normoxia and hypoxia, with CD9 silencing or overexpression. The level of p-Smad2/Smad2 and p-Smad3/Smad3 declined by 43.4% and 52.1% in siCD9 HSFs under hypoxia which indicated that activated TGF-β1/Smad2/3 Signaling was inhibited by silencing CD9. On the contrary, Ad-CD9 HSFs further activated the TGF-β1/Smad2/3 Signaling pathway in hypoxic environment, confirmed by 83.3% and 58.8% higher level of p-Smad2/Smad2 and p-Smad3/Smad3 (Fig. 5C, D). To sum up, CD9 regulated TGF-β1/Smad2/3 Signaling in hypoxic fibroblast through directly affecting TβR1–TβR2 association.

## Discussion

**Fig. 6:**
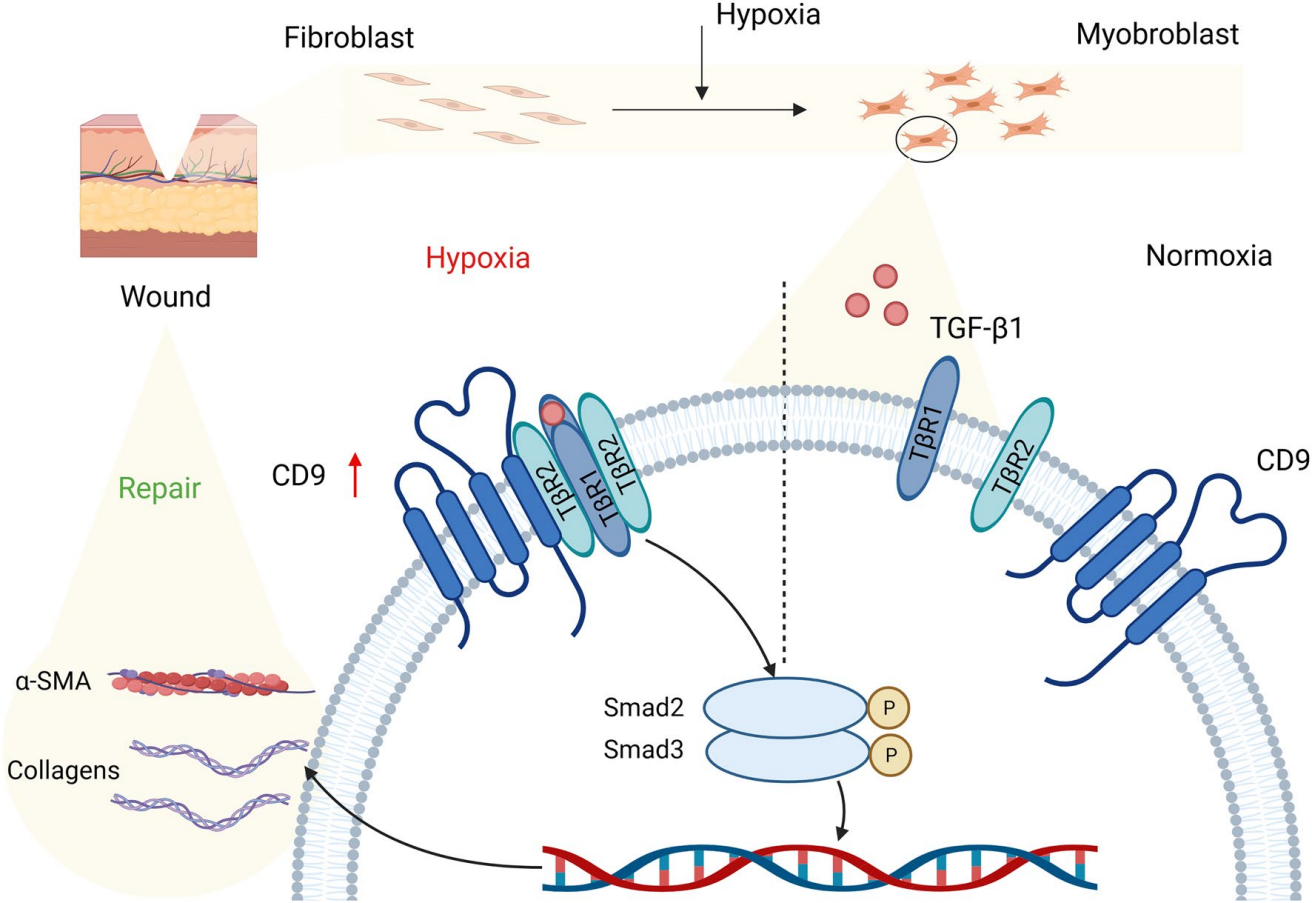
Schematic diagram of CD9 promoting TβR2–TβR1 association, driving the fibroblasts-to-myofibroblast transition under hypoxia

Fibroblast to myofibroblast transition plays a crucial role not only in the pathological fibrosis of organs and tissues, but also in the necessary process of normal skin wound healing (Darby et al. 2016). Recent studies showed that the impaired function of myofibroblasts within wounds may account for chronic and nonhealing wounds (Wall et al. 2008). Although TGF-β1/Smad canonical pathway was involved in fibroblast to myofibroblast transition, its regulatory mechanism in the wound healing process has not yet been fully elucidated (Meng et al. 2016). Through the use of HSFs and mouse skin wounds as models, this study identified that hypoxia directly acted on TβR1 and TβR2 through CD9 to activate the TGF-β1/Smad pathway, which in turn triggered the transition of fibroblasts into myofibroblasts (Fig. 6).

Skin wounds mainly caused by physical damage will lead to compromised vascular integrity and increased cellular requirements for oxygen to initiate the repair process (Younis 2020). Hence, wounds are in a hypoxic environment during the early stages, which can be detected through pimonidazole adduct staining with oxygen tension levels dropping below 10 mmHg (Ninikoski et al. 1971; Goodson et al. 1979; Lokmic et al. 2006). Studies have demonstrated that hypoxia triggered wound healing by promoting vascularization and granulation tissue formation during the early stages of healing (Schreml et al. 2010; Hutami et al. 2021). Despite its importance in the wound healing process, there is still insufficient research on the regulation of fibroblast to myofibroblast transition by hypoxia. In this study, a 2% hypoxic culture model of human skin fibroblasts was established, and it was found that after 12, 24, and 48 h of hypoxia treatment, the expression levels of α-SMA, COL-Ι, and COL-Ш significantly increased, indicating that hypoxia promoted fibroblast to myofibroblast transition. Animal models have also shown the activation of the TGF-β1/Smad pathway under hypoxia in the early stages of wound healing, further confirming the role of hypoxia in inducing fibroblast to myofibroblast transition. Our results are consistent with the study conducted by Zhao et al. which found that 1% hypoxia for 48 h increased the expression of collagens and α-SMA in HSFs by regulating the TGF-β1/Smad3 pathway (Zhao et al. 2017). However, if hypoxia persists over a prolonged period, it can impair the process of fibroblast to myofibroblast transition since Modarressi et al. found a reduction in both the number of myofibroblasts and the secretion of collagens after exposure to 5% and 2% hypoxia for 5 days (Modarressi et al. 2010). One limitation of our study is that α-SMA is the only marker used to distinguish myofibroblasts, so additional techniques such as flow cytometry and single-cell sequencing will be used to identify myofibroblasts more accurately in future studies (Rippa et al. 2019; Chen et al. 2022).

CD9, a member of tetraspanin superfamily, is involved in a range of cellular processes, such as integrin-dependent cell migration, proliferation, and differentiation (Hemler 2014; Machado-Pineda et al. 2018). In our earlier study, we found that CD9 was required in wound healing for delayed wound healing was observed in a CD9 knocking-down mouse model and reduced CD9 was essential for epidermal migration (Zhang et al. 2012; Jiang et al. 2013). However, there is limited research on the effects of CD9 on fibroblast to myofibroblast transition under hypoxia. In this study, we found that CD9 expression was upregulated under hypoxia and CD9 induced fibroblast to myofibroblast transition, which could be reversed by silencing CD9. Consistently, we established a wounded mice model, revealing a significant increase in CD9 expression in the early hypoxic microenvironment, which further confirmed that CD9 played a pivotal role in hypoxia-induced fibroblast-to-myofibroblast transition. Targeting the role of CD9 in wound healing, we previously found that hypoxia-induced CD9 downregulation in keratinocytes contributes to cell migration via P38/ MAPK pathway (Jiang et al. 2014). In addition, Klein-Soyer et al. and Protty et al. has demonstrated that CD9 was involved in the platelet adhesion and migration of endothelial cells, possibly through collaborative interactions with integrins (Klein-Soyer et al. 2000; Protty et al. 2009). Combined with aforementioned research results, we speculate that CD9 plays a crucial role not only in initiating wound healing under early hypoxia microenvironment and but also during the whole process of wound healing.

Tetraspanin proteins is widely expressed on the cell surface, featuring 4 transmembrane domains, along with extracellular and intracellular loops (Hemler 2005; Kovalenko et al. 2005). It plays a crucial role in organizing the cell surface molecules by selectively recruiting partner proteins into tetraspanin-enriched microdomains, allowing for the assembly of a broad range of molecules to amplify their functions (Boucheix et al. 1991; Seigneuret et al. 2001). The association between tetraspanins and transmembrane transforming growth factors has been discovered, revealing that tetraspanins could directly regulated TGF-α-induced EGFR activation and TGF-β/Smads signaling (Wang et al. 2015; Lorico et al. 2021). The TGF-β pathway is widely acknowledged to play a pivotal role in regulating cell proliferation and differentiation, and has been considered as one of the classical pathways controlling the transition of fibroblasts into myofibroblasts (Li et al. 2021). In wound healing, The TGF-β/Smad cascade is activated when TGF-β1 binding to TβR2 and TβR1, phosphorylating cytoplasmic mediators, Smad2 and/or Smad3 (Chen et al. 2018; Zi 2019). Subsequently, Smad2/3 combine with Smad4 to form a heterotrimeric complex that translocate into the nucleus and binds to a specific sequence (Wang et al. 2022). In this study, we found that hypoxia could activate the TGFβ1 pathway, as evidenced by higher levels of p-Smad2/3 in both in vitro and vivo. Notably, hypoxia does not affect the expression levels of TβR1 and TβR2 in the cell membrane or cytoplasm, but significantly affects their interaction. Further studies have confirmed that knocking down CD9 under hypoxia reduces the interaction between TβR1 and TβR2, as well as higher levels of p-Smad2/3, while overexpression of CD9 has the opposite effect. Therefore, we speculate that hypoxia directly activates the TGFβ1/Smad pathway by promoting TβR1 and TβR2 via CD9, thereby mediating the transition of fibroblasts into myofibroblasts. Moreover, the level of TGF-β1 under normoxia and hypoxia was measured by ELISA, and it was found that hypoxia had no significant effect on the level of TGF-β1 (Figure S1A). Therefore, hypoxia does not cause an increase in the level of TGF-β1 to generate an autocrine loop. Then, we examined the production of TGF-β1 when knocking down or overexpressing CD9, and also found that there was still no significant difference in the level of TGF-β1 (Figure S1C, D). This further supports our conclusion that CD9 acts directly on TβR1 and TβR2 that phosphorylate the intracellular Smad proteins as an organizer under hypoxia, which is also consistent with reports in previous research (Mallikarjuna et al. 2022). Additionally, we discovered that hypoxia upregulates CD9 expression in human keloid fibroblasts, so further research is necessary to explore if tetraspanins mediate scar formation under hypoxic conditions, given the fact that TGF-β1/Smad pathway promotes scars (Ong et al. 2021).

Overall, we observed that CD9 promotes TβR2–TβR1 association under hypoxia, which promotes fibroblast to myofibroblast transition in both hypoxic HSFs and wounded animal models. These results provide a new understanding of the role of tetraspanin CD9 in regulating wound healing and the TGF-β1/Smad pathway-induced transition of fibroblasts to myofibroblasts during the initial wound healing phases under physiological hypoxia.

## Supplementary Information

Supplementary Material 1.

Supplementary Material 2.

## Data Availability

The data used to support the findings of this study are available from the corresponding author upon reasonable request.

## Abbreviations

HSFs: Human skin fibroblasts
HKFs: Human keloid fibroblasts
CCK8: Cell Counting Kit-8
COL-Ι: Type I fibrillar collagens
COL-Ш: Type Ш fibrillar collagens
α-SMA: α-Smooth muscle actin
ECM: Extracellular matrix
TGF-β1: Transforming growth factor beta 1
TβR1: TGF-β type I receptors
TβR2: TGF-β type II receptors
EMT: Epithelial-mesenchymal
IHC: Immunohistochemistry
IF: Immunofluorescence
IP: Immunoprecipitation
SEM: Standard error of mean
CK10: Cytokeratin 10

## References

[CR1] Boucheix C, Benoit P, Frachet P, Billard M, Worthington RE, Gagnon J, et al. Molecular cloning of the CD9 antigen. A new family of cell surface proteins. J Biol Chem. 1991;266:117–22. 1840589

[CR2] Brosseau C, Colas L, Magnan A, Brouard S. CD9 tetraspanin: a new pathway for the regulation of inflammation? Front Immunol. 2018;9:2316. 30356731 10.3389/fimmu.2018.02316 PMC6189363

[CR3] Caja L, Dituri F, Mancarella S, Caballero-Diaz D, Moustakas A, Giannelli G, et al. TGF-β and the tissue microenvironment: relevance in fibrosis and cancer. Int J Mol Sci. 2018;19:1294. 29701666 10.3390/ijms19051294 PMC5983604

[CR4] Carthy JM. TGFβ signaling and the control of myofibroblast differentiation: Implications for chronic inflammatory disorders. J Cell Physiol. 2018;233:98–106. 28247933 10.1002/jcp.25879

[CR5] Castilho RM, Squarize CH, Gutkind JS. Exploiting PI3K/mTOR signaling to accelerate epithelial wound healing. Oral Dis. 2013;19:551–8. 23379329 10.1111/odi.12070 PMC4764999

[CR6] Chen L, Yang T, Lu DW, Zhao H, Feng YL, Chen H, et al. Central role of dysregulation of TGF-β/Smad in CKD progression and potential targets of its treatment. Biomed Pharmacother. 2018;101:670–81. 29518614 10.1016/j.biopha.2018.02.090

[CR7] Chen CJ, Kajita H, Takaya K, Aramaki-Hattori N, Sakai S, Asou T, et al. Single-Cell RNA-seq analysis reveals cellular functional heterogeneity in dermis between fibrotic and regenerative wound healing fates. Front Immunol. 2022;13: 875407. 35664010 10.3389/fimmu.2022.875407 PMC9156976

[CR8] Darby IA, Hewitson TD. Hypoxia in tissue repair and fibrosis. Cell Tissue Res. 2016;365:553–62. 27423661 10.1007/s00441-016-2461-3

[CR9] Darby IA, Zakuan N, Billet F, Desmoulière A. The myofibroblast, a key cell in normal and pathological tissue repair. Cell Mol Life Sci. 2016;73:1145–57. 26681260 10.1007/s00018-015-2110-0 PMC11108523

[CR10] Distler JHW, Györfi AH, Ramanujam M, Whitfield ML, Königshoff M, Lafyatis R. Shared and distinct mechanisms of fibrosis. Nat Rev Rheumatol. 2019;15:705–30. 31712723 10.1038/s41584-019-0322-7

[CR11] Goodson WH, Andrews WS, Thakral KK, Hunt TK. Wound oxygen tension of large vs small wounds in man. Surg Forum. 1979;30:92–5. 538708

[CR12] Hemler ME. Tetraspanin functions and associated microdomains. Nat Rev Mol Cell Biol. 2005;6:801–11. 16314869 10.1038/nrm1736

[CR13] Hemler ME. Tetraspanin proteins promote multiple cancer stages. Nat Rev Cancer. 2014;14:49–60. 24505619 10.1038/nrc3640

[CR14] Hinz B, Mastrangelo D, Iselin CE, Chaponnier C, Gabbiani G. Mechanical tension controls granulation tissue contractile activity and myofibroblast differentiation. Am J Pathol. 2001;159:1009–20. 11549593 10.1016/S0002-9440(10)61776-2 PMC1850455

[CR15] Hong WX, Hu MS, Esquivel M, Liang GY, Rennert RC, McArdle A, et al. The role of hypoxia-inducible factor in wound healing. Adv Wound Care (New Rochelle). 2014;3:390–9. 24804159 10.1089/wound.2013.0520 PMC4005494

[CR16] Hutami IR, Izawa T, Khurel-Ochir T, Sakamaki T, Iwasa A, Tanaka E. Macrophage motility in wound healing is regulated by HIF-1α via S1P signaling. Int J Mol Sci. 2021;22:8992. 34445695 10.3390/ijms22168992 PMC8396560

[CR17] Jiang XP, Zhang DX, Teng M, Zhang Q, Zhang JP, Huang YS. Downregulation of CD9 in keratinocyte contributes to cell migration via upregulation of matrix metalloproteinase-9. PLoS ONE. 2013;8: e77806. 24147081 10.1371/journal.pone.0077806 PMC3797697

[CR18] Jiang X, Guo X, Xu X, Teng M, Huang C, Zhang D, et al. Hypoxia regulates CD9-mediated keratinocyte migration via the P38/MAPK pathway. Sci Rep. 2014;4:6304. 25200404 10.1038/srep06304 PMC4158574

[CR19] Jiang X, Teng M, Ji R, Zhang D, Zhang Z, Lv Y, et al. CD9 regulates keratinocyte differentiation and motility by recruiting E-cadherin to the plasma membrane and activating the PI3K/Akt pathway. Biochim Biophys Acta Mol Cell Res. 2020;1867: 118574. 31682865 10.1016/j.bbamcr.2019.118574

[CR20] Klein-Soyer C, Azorsa DO, Cazenave JP, Lanza F. CD9 participates in endothelial cell migration during in vitro wound repair. Arterioscler Thromb Vasc Biol. 2000;20:360–9. 10669631 10.1161/01.atv.20.2.360

[CR21] Kovalenko OV, Metcalf DG, DeGrado WF, Hemler ME. Structural organization and interactions of transmembrane domains in tetraspanin proteins. BMC Struct Biol. 2005;5:11. 15985154 10.1186/1472-6807-5-11 PMC1190194

[CR22] Leinhos L, Peters J, Krull S, Helbig L, Vogler M, Levay M, et al. Hypoxia suppresses myofibroblast differentiation by changing RhoA activity. J Cell Sci. 2019;132:jcs223230. 30659117 10.1242/jcs.223230

[CR23] Li X, Ding Z, Wu Z, Xu Y, Yao H, Lin K. Targeting the TGF-β signaling pathway for fibrosis therapy: a patent review (2015–2020). Expert Opin Ther Pat. 2021;31:723–43. 33645365 10.1080/13543776.2021.1896705

[CR24] Liu L, Sun Q, Davis F, Mao J, Zhao H, Ma D. Epithelial-mesenchymal transition in organ fibrosis development: current understanding and treatment strategies. Burns Trauma. 2022;10:tkac011. 35402628 10.1093/burnst/tkac011 PMC8990740

[CR25] Lokmic Z, Darby IA, Thompson EW, Mitchell GM. Time course analysis of hypoxia, granulation tissue and blood vessel growth, and remodeling in healing rat cutaneous incisional primary intention wounds. Wound Repair Regen. 2006;14:277–88. 16808806 10.1111/j.1743-6109.2006.00122.x

[CR26] Lorico A, Lorico-Rappa M, Karbanová J, Corbeil D, Pizzorno G. CD9, a tetraspanin target for cancer therapy? Exp Biol Med (Maywood). 2021;246:1121–38. 33601913 10.1177/1535370220981855 PMC8113732

[CR27] Machado-Pineda Y, Cardeñes B, Reyes R, López-Martín S, Toribio V, Sánchez-Organero P, et al. CD9 controls integrin α5β1-mediated cell adhesion by modulating its association with the metalloproteinase ADAM17. Front Immunol. 2018;9:2474. 30455686 10.3389/fimmu.2018.02474 PMC6230984

[CR28] Mallikarjuna P, Zhou Y, Landström M. The synergistic cooperation between TGF-β and hypoxia in cancer and fibrosis. Biomolecules. 2022;12:635. 35625561 10.3390/biom12050635 PMC9138354

[CR29] Meng XM, Nikolic-Paterson DJ, Lan HY. TGF-β: the master regulator of fibrosis. Nat Rev Nephrol. 2016;12:325–38. 27108839 10.1038/nrneph.2016.48

[CR30] Mingyuan X, Qianqian P, Shengquan X, Chenyi Y, Rui L, Yichen S, et al. Hypoxia-inducible factor-1α activates transforming growth factor-β1/Smad signaling and increases collagen deposition in dermal fibroblasts. Oncotarget. 2018;9:3188–97. 29423039 10.18632/oncotarget.23225 PMC5790456

[CR31] Miyazawa K, Miyazono K. Regulation of TGF-β family signaling by inhibitory smads. Cold Spring Harb Perspect Biol. 2017;9: 022095. 10.1101/cshperspect.a022095 PMC5334261 27920040

[CR32] Modarressi A, Pietramaggiori G, Godbout C, Vigato E, Pittet B, Hinz B. Hypoxia impairs skin myofibroblast differentiation and function. J Invest Dermatol. 2010;130:2818–27. 20686497 10.1038/jid.2010.224

[CR33] Ninikoski J, Heughan C, Hunt TK. Oxygen and carbon dioxide tensions in experimental wounds. Surg Gynecol Obstet. 1971;133:1003–7. 5117384

[CR34] Ong CH, Tham CL, Harith HH, Firdaus N, Israf DA. TGF-β-induced fibrosis: A review on the underlying mechanism and potential therapeutic strategies. Eur J Pharmacol. 2021;911: 174510. 34560077 10.1016/j.ejphar.2021.174510

[CR35] Orenstein JM. “myofibroblast” that is omnipresent in pathology and key to the EMT concepts does not actually exist, since normal fibroblasts contain stress fibril organelles (SMA bundles with dense bodies) variably detected by TEM and IHC: conclusions by a diagnostic pathologist with decades of ultrastructural experience. Ultrastruct Pathol. 2014;38:387–98. 25084158 10.3109/01913123.2014.940231

[CR36] Oritani K, Aoyama K, Tomiyama Y, Kincade PW, Matsuzawa Y. Stromal cell CD9 and the differentiation of hematopoietic stem/progenitor cells. Leuk Lymphoma. 2000;38:147–52. 10811457 10.3109/10428190009060328

[CR37] Protty MB, Watkins NA, Colombo D, Thomas SG, Heath VL, Herbert JM, et al. Identification of Tspan9 as a novel platelet tetraspanin and the collagen receptor GPVI as a component of tetraspanin microdomains. Biochem J. 2009;417:391–400. 18795891 10.1042/BJ20081126 PMC2652832

[CR38] Rippa AL, Kalabusheva EP, Vorotelyak EA. Regeneration of dermis: scarring and cells involved. Cells. 2019;8:607. 31216669 10.3390/cells8060607 PMC6627856

[CR39] Rodrigues M, Kosaric N, Bonham CA, Gurtner GC. Wound healing: a cellular perspective. Physiol Rev. 2019;99:665–706. 30475656 10.1152/physrev.00067.2017 PMC6442927

[CR40] Schreml S, Szeimies RM, Prantl L, Karrer S, Landthaler M, Babilas P. Oxygen in acute and chronic wound healing. Br J Dermatol. 2010;163:257–68. 20394633 10.1111/j.1365-2133.2010.09804.x

[CR41] Seigneuret M, Delaguillaumie A, Lagaudrière-Gesbert C, Conjeaud H. Structure of the tetraspanin main extracellular domain. A partially conserved fold with a structurally variable domain insertion. J Biol Chem. 2001;276:40055–64. 11483611 10.1074/jbc.M105557200

[CR42] Suttho D, Mankhetkorn S, Binda D, Pazart L, Humbert P, Rolin G. 3D modeling of keloid scars in vitro by cell and tissue engineering. Arch Dermatol Res. 2017;309:55–62. 27942931 10.1007/s00403-016-1703-2

[CR43] Tai Y, Woods EL, Dally J, Kong D, Steadman R, Moseley R, et al. Myofibroblasts: function, formation, and scope of molecular therapies for skin fibrosis. Biomolecules. 2021;11:1095. 34439762 10.3390/biom11081095 PMC8391320

[CR44] Tirpe AA, Gulei D, Ciortea SM, Crivii C, Berindan-Neagoe I. Hypoxia: overview on hypoxia-mediated mechanisms with a focus on the role of HIF genes. Int J Mol Sci. 2019;20:6140. 31817513 10.3390/ijms20246140 PMC6941045

[CR45] Wall IB, Moseley R, Baird DM, Kipling D, Giles P, Laffafian I, et al. Fibroblast dysfunction is a key factor in the non-healing of chronic venous leg ulcers. J Invest Dermatol. 2008;128:2526–40. 18449211 10.1038/jid.2008.114

[CR46] Wang HX, Sharma C, Knoblich K, Granter SR, Hemler ME. EWI-2 negatively regulates TGF-β signaling leading to altered melanoma growth and metastasis. Cell Res. 2015;25:370–85. 25656846 10.1038/cr.2015.17 PMC4349253

[CR47] Wang Q, Wang P, Qin Z, Yang X, Pan B, Nie F, et al. Altered glucose metabolism and cell function in keloid fibroblasts under hypoxia. Redox Biol. 2021;38: 101815. 33278780 10.1016/j.redox.2020.101815 PMC7718484

[CR48] Wang J, Shang R, Yang J, Liu Z, Chen Y, Chen C, et al. P311 promotes type II transforming growth factor-β receptor mediated fibroblast activation and granulation tissue formation in wound healing. Burns Trauma. 2022;10:tkac027. 37469904 10.1093/burnst/tkac027 PMC9562783

[CR49] Xing D, Liu L, Marti GP, Zhang X, Reinblatt M, Milner SM, et al. Hypoxia and hypoxia-inducible factor in the burn wound. Wound Repair Regen. 2011;19:205–13. 21362088 10.1111/j.1524-475X.2010.00656.x PMC3075089

[CR50] Younis I. Role of oxygen in wound healing. J Wound Care. 2020;29:S4-s10. 32427027 10.12968/jowc.2020.29.Sup5b.S4

[CR51] Zhang J, Dong J, Gu H, Yu S, Zhang X, Gou Y, et al. CD9 is critical for cutaneous wound healing through JNK signaling. J Invest Dermatol. 2012;132:226–36. 21881583 10.1038/jid.2011.268

[CR52] Zhao B, Guan H, Liu JQ, Zheng Z, Zhou Q, Zhang J, et al. Hypoxia drives the transition of human dermal fibroblasts to a myofibroblast-like phenotype via the TGF-β1/Smad3 pathway. Int J Mol Med. 2017;39:153–9. 27909731 10.3892/ijmm.2016.2816 PMC5179176

[CR53] Zi Z. Molecular engineering of the TGF-β signaling pathway. J Mol Biol. 2019;431:2644–54. 31121181 10.1016/j.jmb.2019.05.022

