# Supplementary material for: CD9 promotes TβR2–TβR1 association driving the transition of human dermal fibroblasts to myofibroblast under hypoxia
Source: Mol Med. 2024 Sep 27;30:162. doi: 10.1186/s10020-024-00925-5 (PMC11428569; doi:10.1186/s10020-024-00925-5)
Supplement: Supplementary file 1 — Supplementary Material 1. [file 10020_2024_925_MOESM1_ESM.docx]

***Supplementary Material***

**CD9 promotes TβR2-TβR1 association driving the transition of human dermal fibroblasts to myofibroblast under hypoxia**

**Materials and Methods**

**Detection of the level of TGF-β1 by ELISA**

The level of TGF-β1 release was quantified with ELISA as directed by the manufacturer. Briefly, HSF cells were grown in six-well plate to 80% density, and then cells were under normoxia or hypoxia for 24h. After that, the cultures up supernatant was removed and evaluated for TGF-β1 content using the corresponding ELISA kit (EK981-48, Multi Sciences, China).

**Supporting figure captions:**

**Figure S1**: The level of TGF-β1 in HSF supernatant detected by ELISA in different conditions. (A) The level of TGF-β1 in HSF supernatant under normoxic and hypoxic conditions. (B) The level of TGF-β1 in HSF supernatant under normoxia and under hypoxia when silencing CD9 (siCD9) in HSFs. (C) The level of TGF-β1 in HSF supernatant under normoxia and under hypoxia when overexpressing CD9 (Ad-CD9) in HSFs.
