# Supplementary figures and images for: CD9 promotes TβR2–TβR1 association driving the transition of human dermal fibroblasts to myofibroblast under hypoxia
Source: Mol Med. 2024 Sep 27;30:162. doi: 10.1186/s10020-024-00925-5 (PMC11428569; doi:10.1186/s10020-024-00925-5)

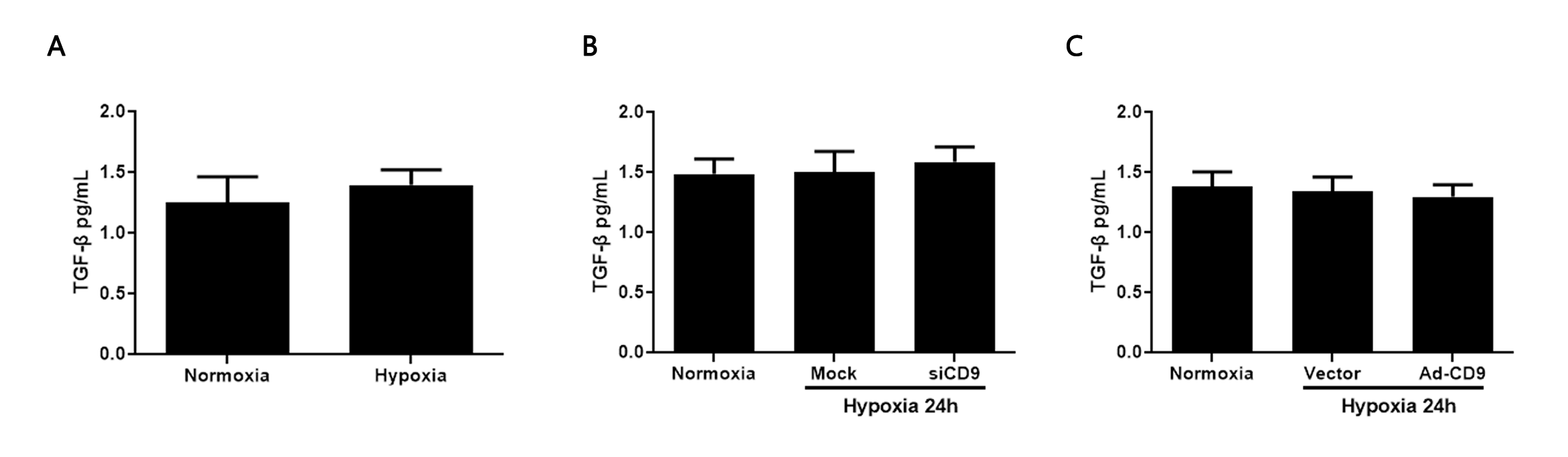

Supplement: Supplementary file 2 — Supplementary Material 2. [file 10020_2024_925_MOESM2_ESM.tif]
